# Pilot study evaluating the effects of an intervention to enhance culturally appropriate hypertension education among healthcare providers in a primary care setting

**DOI:** 10.1186/1748-5908-5-35

**Published:** 2010-05-14

**Authors:** Erik JAJ Beune, Patrick JE Bindels, Jacob Mohrs, Karien Stronks, Joke A Haafkens

**Affiliations:** 1 Department of General Practice/Clinical Methods and Public Health, Academic Medical Centre, University of Amsterdam, Meibergdreef 15, Amsterdam, The Netherlands; 2 Department of General Practice, Erasmus MC, Burg. s' Jacobplein 51, 3015 CA Rotterdam, The Netherlands; 3 Department of Social Medicine/Clinical Methods and Public Health, Academic Medical Centre, University of Amsterdam, Meibergdreef 15, Amsterdam, The Netherlands

## Abstract

**Background:**

To improve hypertension care for ethnic minority patients of African descent in the Netherlands, we developed a provider intervention to facilitate the delivery of culturally appropriate hypertension education. This pilot study evaluates how the intervention affected the attitudes and perceived competence of hypertension care providers with regard to culturally appropriate care.

**Methods:**

Pre- and post-intervention questionnaires were used to measure the attitudes, experienced barriers, and self-reported behaviour of healthcare providers with regard to culturally appropriate cardiovascular and general care at three intervention sites (N = 47) and three control sites (N = 35).

**Results:**

Forty-nine participants (60%) completed questionnaires at baseline (T0) and nine months later (T1). At T1, healthcare providers who received the intervention found it more important to consider the patient's culture when delivering care than healthcare providers who did not receive the intervention (p = 0.030). The intervention did not influence experienced barriers and self-reported behaviour with regard to culturally appropriate care delivery.

**Conclusion:**

There is preliminary evidence that the intervention can increase the acceptance of a culturally appropriate approach to hypertension care among hypertension educators in routine primary care.

## Background

In Western countries, ethnic minority populations of African descent have higher rates of hypertension and worse hypertension-related health outcomes than Europeans [1-3].

This has also been observed among Afro-Surinamese (hereafter, Surinamese) and Ghanaians living in the Netherlands. A recent study conducted in Amsterdam reported a higher prevalence of hypertension in Surinamese (47%) than in ethnically Dutch people (33%). Treatment rates were the same for both groups, but Surinamese who were treated for hypertension had lower rates of blood pressure control [4], which may explain the excess mortality due to stroke found in this group [5]. Hypertension is also highly prevalent among Ghanaians [6,7].

Poor adherence to antihypertensive medication and therapeutic lifestyle changes is an important modifiable factor contributing to ethnic disparities in blood pressure control [8,9]. There is evidence that patients' health beliefs can be an important barrier to adherence [10-12], and that culture can influence those beliefs [13-17]. This was also found in our own studies of Surinamese, Ghanaian, and ethnically Dutch hypertensive patients living in the Netherlands [18-20].

Hypertension guidelines recommend patient education as a tool for improving adherence [21,22]. There is some evidence that culturally appropriate educational interventions can improve treatment outcomes in ethnic minority patients [23,24]. However, the literature provides no descriptions of those interventions for hypertensive patients [25,26].

For this reason, we developed an intervention to facilitate the delivery of culturally appropriate hypertension education (CAHE) by primary care providers. In a previous study, we identified two barriers that may prevent healthcare providers from using CAHE: a negative attitude towards culturally appropriate care in general and a lack of the skills needed to implement this type of health education [27]. Thus, we conducted a pilot with the aim of evaluating whether the intervention could remove these barriers.

## Methods

### Study design, setting, and participants

**Figure 1:**
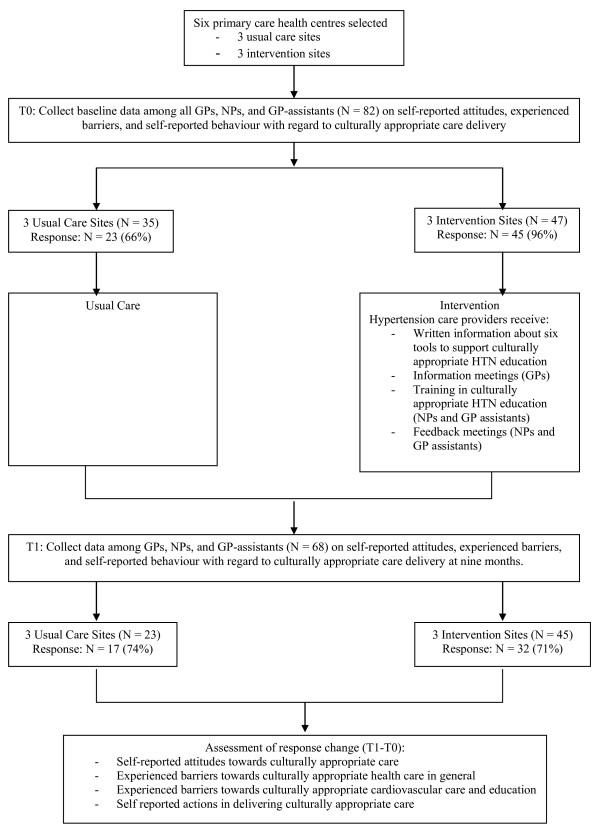
**Overview of the implementation and the measurement**.

We used a quasi-experimental design, contrasting intervention and control groups, to evaluate the effects of the intervention (see Figure 1). The study was conducted in six primary care health centres (PCHCs) belonging to the GAZO healthcare consortium in southeast Amsterdam. This area was chosen because it has a relatively high proportion of Surinamese and Ghanaian residents. Three of the selected PCHCs had also participated in a previous study [18-20,27], and they volunteered to pilot the intervention. The three other PCHCs served as control centres. It is estimated that 26% of the 24,094 patients registered in the intervention centres and 26% of the 20,076 patients registered in the control centres are of Surinamese or Ghanaian origin (data are from 2007). Based on data from the SUNSET study [4], we expected that some 47% of the patients of African origin would suffer from hypertension.

All six centres used a similar protocol for hypertension care, based on the guidelines of the Dutch College of General Practitioners [21]. According to this protocol, hypertension education for patients with uncomplicated hypertension can be provided by a general practitioner (GP), a nurse practitioner (NP), or a general practice assistant (GP assistant) under the supervision of a GP. The intervention targeted all healthcare providers who provide hypertension education to patients with uncomplicated hypertension (K86). The intervention group consisted of 47 healthcare providers: 7 NPs, 18 GP assistants and 22 GPs. The control group consisted of 35 healthcare providers: 5 NPs, 14 GP assistants and 16 GPs.

### Intervention

The aim of the intervention was to support healthcare providers in using CAHE, specifically for Surinamese and Ghanaian patients. Interventions are more likely to elicit change in healthcare professionals if they use multiple approaches [28,29]. Our intervention consisted of three components: written tools, training, and feedback.

### Written tools

We supplemented the standard hypertension protocol used by the intervention centres with information about six tools to support CAHE:

1. A topic list to explore the patient's ideas, concerns, and expectations regarding hypertension and hypertension treatment.

2. A topic list to explore culturally specific barriers to and facilitators of treatment adherence. The items on the lists were derived from the work of Kleinman [30,31], recent approaches to improve adherence [10,32,33], and our prior study [18-20] (see Table 1).

**Table 1:** Topic list for eliciting immigrant patients' explanatory model of hypertension^1^

Communication Determine how a patient wants to be addressed (formally or informally). Determine the patient's preferred language for speaking and reading (Dutch or another language). Use this information in your interaction with the patient.
**Introduction** It is often difficult for us (care providers) to give advice about hypertension and how to manage it if we are not familiar with the views and experiences of our patients. For that reason, I would like to ask you some questions to learn more about your own views on hypertension and its treatment.
**Topic list one: Elicit personal views on hypertension and its treatment**
**Understanding** What do you understand hypertension to mean? **Causes** What do you think has caused your hypertension? Why did it occur now/when it did; why to you? **Meaning and symptoms** What does it mean to you to have hypertension? Do you notice anything about your hypertension? How do you react in this case? **Duration and consequences** How do you think your hypertension will develop further? How severe is it? What consequences do you think your hypertension may have for you (physical, psychological, social)? **Treatment** What types of treatment do you think would be useful? What does the prescribed therapeutic measurement(s) mean to you?
**Topic list two: Elicit contextual influences on hypertension management**
**Social** Do you speak with family/community members about your hypertension? How do they react? Do family/community members help you or make it difficult for you to manage hypertension? Please explain.
**Culture/religion** Are there any cultural issues/religious issues that may help you or make it difficult for you to manage hypertension? Please explain.
**Migration** Are there any issues related to your position as an immigrant that make it difficult to you to manage hypertension? Please explain.
**Finance** Are there any issues related to your financial situation that make it difficult for you to manage hypertension? Please explain.

^1^Based on Kleinman's Explanatory Model format [30,31] and our previous study [18-20].

3. A checklist to facilitate the recognition of specific barriers to hypertension management in Surinamese and Ghanaian patients, based on our prior study [18-20].

4. Information leaflets for Surinamese or Ghanaian patients with answers to frequently asked questions about hypertension. These leaflets were adapted to the language, customs, habits, norms, and dietary cultures of the Surinamese and Ghanaian communities, using information obtained from our previous study [18-20]. Consideration was also given to recommended surface and deep structure elements [34]. The leaflets were pre-tested in two focus groups with Surinamese and Ghanaian hypertensive patients.

5. A referral list, including neighbourhood facilities offering healthier lifestyle support tailored to Surinamese and Ghanaian patients.

6. A list of items used to register the results of hypertension counselling sessions.

Information about these tools was made available on paper and also through pop-up screens in the digital hypertension protocol used by the intervention centres.

### Training and feedback

To support the use of these tools, we provided a training course of two half-day sessions to all NPs and GP assistants in the intervention centres. During the first session, information about the prevalence and treatment of hypertension among populations of African origin in Western countries was provided and discussed. There was also discussion of how the tools might be used. During the second session, training was given in culturally sensitive counselling skills through role-playing exercises with Surinamese and Ghanaian hypertensive patients. Educational materials consisted of a course manual and instruction on the use of the new tools. As a second supportive intervention the researcher (EB) organised feedback meetings (lasting 1.5 hours) with the NPs and GP assistants once every two months.

NPs and GP assistants could also ask for individual advice. The GPs were invited to an information meeting at their health centre (lasting one hour) at the start of the project and received feedback after every group meeting with the NPs and GP assistants.

### Implementation of the intervention

Implementation started in April 2007 with the training course for NPs and GP assistants. The GPs were not invited because almost all of them had completed a somewhat similar training, organised by the PCHCs at an earlier stage. After the training, the tools for CAHE were made available on paper to the healthcare providers. Two and four months later, these tools could also be accessed through the digital hypertension protocol on the PCHC intranet portal. Technical circumstances delayed the intranet access to this protocol. During follow-up, five information meetings for GPs and seven feedback meetings for NPs and GP assistants were held and individual coaching sessions on request.

### Measurement

A questionnaire was used to evaluate the extent to which the intervention had been able to remove previously observed barriers to the provision of culturally appropriate hypertension care (negative attitudes and a lack of perceived competence). We used the 'Resident Physicians' Preparedness to Provide Cross-Cultural Care' survey for this purpose [35]. This instrument was used previously to measure effects of cross-cultural training among physicians in academic health centres. It measures attitudes and perceived competence with regard to culturally appropriate healthcare in general. Because we were particularly interested in cardiovascular care, we adapted this instrument for the purpose of our study. Our questionnaire consisted of four scales. Each scale contains a number of items (questions) to measure a single construct. Scale one measures attitudes towards delivering culturally appropriate care (six items), scale two measures the experienced barriers to the delivery of culturally appropriate care in general (nine items), scale three measures the experienced barriers to the delivery of culturally appropriate cardiovascular care and education (eight items), and scale four measures the self-reported actions in delivering culturally appropriate care (17 items). Respondents had to answer the questions by picking a response option on a four- or five-point Likert scale, which is a commonly used instrument in psychological research on attitudes and self-reported behaviours.

Measurements were performed in April 2007 before the training course was given (T0), and nine months later (T1). On both occasions, the questionnaires were distributed with an explanatory covering letter. Reminders were sent two and four weeks later.

### Data analysis

Completed questionnaires were entered into SPSS Data Entry 4.0 (Ref: SPSS Inc, Chicago IL, USA) and checked for errors using a random test. A first analysis of the data revealed that some of the questions included in the questionnaire could not be answered by NPs and GP assistants, because they were not applicable to their work (three, three, and two items of scales two, three, and four, respectively). This could be explained by the fact that the original instrument had only been tested among physicians, but not among nurses. These items were removed. With the remaining items, we reconstructed the four scales of the questionnaire, using principal component analysis. These scales were consistent and, based on Cronbach's alpha scores, the psychometric characteristics of the scales were good (see Table 2).

**Table 2:** Components and psychometric properties of the questionnaire after scale construction

Sections	Items	Response options	Item total scores	*Internal consistency
(1) Attitude towards delivering culturally appropriate care	How important do you consider the patient's culture to be when providing care: (a) to those from cultures different from your own? (b) to those with health beliefs or practices at odds with Western medicine? (c) to those who distrust the Dutch healthcare system? (d) to those who are members of ethnic minorities? (e) to those whose religious beliefs affect treatment?	1. not at all 2. not very 3. somewhat 4. fairly 5. extremely	5 10 15 20 25	0.871

(2) Experienced barriers to the delivery of culturally appropriate care in general	How often during your work have you experienced cross-cultural or language barriers that led to: (a) unnecessary encounters? (b) unnecessarily long duration of treatment? (c) difficulties with lifestyle counselling? (d) patients' nonadherence? (e) erosion of quality of care?	1. never 2. rarely 3. often 4. always	5 10 15 20	0.800

(3) Experienced barriers to the delivery of culturally appropriate cardiovascular care and education	How much of a problem do you consider each of the following to be when you provide cardiovascular care and education to patients of different cultural backgrounds? (a) Lack of practical experience in caring for ethnic minority patients. b) Lack of time to adequately address immigration and culture-related aspects. (c) Lack of training in culturally appropriate health education in cardiovascular care. (d) Lack of information about culturally sensitive health education in the cardiovascular protocols used in routine practice.	1. no problem 2. small problem 3. moderate problem 4. big problem	4 8 12 16	0.803

(4) Self-reported actions in delivering culturally appropriate care	How often do you consider a patient's cultural background while: (a) determining how a patient wants to be addressed and interacted with? (b) performing an anamnesis? (c) eliciting patients' understanding of illness? (d) eliciting patients' perceptions regarding prescribed medication? (e) eliciting patients' perceptions regarding required lifestyle change? (f) identifying patients' customs that might affect adherence to clinical care? (g) assessing the influence of family or community members on adherence to clinical care?	1. never 2. rarely 3. often 4. always	7 14 21 28	0.865


* Cronbach's alpha

To reduce the effect of confounding factors, the final data analysis was only based on observations from participants who completed the questionnaires twice, at T0 and at T1.

To review response changes, we computed the mean scores and standard deviations of the respondents at T0 and T1 for each of the four scales. Differences in scores between the intervention and the control groups at T0 and T1 were tested using one-way analysis of variance for the four scales. To correct for confounding effect of the higher baseline scores of the intervention group at scale four, an additional regression analysis was performed. Test-statistics with a p-value of less than 0.05 were considered statistically significant. All statistical analyses were performed using SPSS version 16.0 (SPSS Inc, Chicago IL, USA).

### Ethics

The study protocol was submitted to the Medical Ethical Committee of the Academic Medical Centre of the University of Amsterdam. The Committee established that the study does not fall within the realm of the Dutch Law Medical Scientific Research with humans because it does not include a medical intervention or invasive measures with humans. For that reason, the Committee sent a letter stating that the study does not require further assessment and approval from the Medical Ethical Committee of the Academic Medical Centre (AMC) of the University of Amsterdam or from any other officially accredited Medical Ethical Research Committee in the Netherlands (reference number 09171260). However, in line with the AMC code for the good conduct of medical research [36], provisions were made to assure the respondents anonymity in collection, analysis, and presentation of the data.

## Results

All but two of the 25 invited NPs and GP assistants (92%) from the intervention PCHCs attended the training course. After the training course, 18 of the 22 GPs in the intervention group (82%) attended information meetings; 16 of the 25 NPs and GP assistants (64%) attended feedback meetings and seven of them (28%) had asked for individual coaching sessions.

A total of 82 questionnaires were sent out at baseline (T0), 47 to the intervention group and 35 to the control group. Forty-nine participants (60%) completed the questionnaires both at baseline (T0) and nine months later (T1), 32 (68%) in the intervention group and 17 (49%) in the control group.

The characteristics of the respondents are displayed in Table 3. The mean age of those who completed both questionnaires was 47 years, the majority were female (80%) and had a Dutch ethnic background (81%). These characteristics did not differ much between the intervention and control groups.

**Table 3:** Characteristics of respondents to questionnaires at T0 and T1: intervention and control groups

Characteristic	Intervention (N = 32)	Control (N = 17)
**Age**		

Mean (sd)	49.5 (8.6)	44.3 (11.7)

**Gender**		

- Male: N (%)	7 (22)	3 (18)

- Female: N (%)	25 (78)	14 (82)

**Ethnicity**		

- Dutch: N (%)	26 (81)	15 (88)

- Other*: N (%)	6 (19)	2 (12)

**Profession**		

- GP: N (%)	16 (50)	9 (53)

- NP: N (%)	5 (16)	3 (18)

- GP ass: N (%)	11 (34)	5 (29)

*Self or minimally one parent born outside the Netherlands

Table 4 shows the mean scores of the respondents of the intervention and control groups and the results of the ANOVA analysis on each of the four scales at T0 and at T1. At baseline, no significant differences were found between both groups with respect to the attitudes towards culturally appropriate care (scale one) and the perceived barriers for delivering it (scale two and three). The baseline scores on scale four were significantly higher in the intervention group compared to the control group (p = 0.012). This indicates that, at the start of the project, the intervention group more often considered a patient's cultural background while delivering care than the control group. At T1, healthcare providers who received the intervention found it more important to consider the patient's culture when delivering care than healthcare providers who did not receive the intervention (scale one, p = 0.030). No significant differences were found for: scale two, experienced barriers in delivering culturally appropriate care in general; scale three, experienced barriers towards culturally appropriate cardiovascular care and education; and scale four, self-reported culturally appropriate healthcare behaviour. Because the higher baseline scores on scale four at T0 in the intervention group might be a confounder, we have corrected for this variable in an additional regression-analysis. After this correction, the important and significant effect from the intervention on 'scale one: attitude towards culturally appropriate care' at T1 remained and was even stronger (p = 0.013).

**Table 4:** Comparison of the intervention and control groups at T0 and at T1

One-way ANOVA for the four scales					
	**Intervention**	**Control**			
	
	**Mean (SD)**	**Mean (SD)**	**df**	**F**	**p-value**

Scale one T0	19.000 (4.348)	17.647 (2.914)	48	1.323	0.256

Scale one T1	20.156 (3.602)	17.765 (3.501)	48	4.980	0.030*

Scale two T0	7.625 (1.548)	7.714 (1.541)	42	0.066	0.798

Scale two T1	8.037 (1.018)	7.643 (1.277)	42	0.446	0.508

Scale three T0	11.13 (2.581)	10.833 (1.992)	37	0.196	0.661

Scale three T1	9.783 (2.696)	9.417 (2.811)	40	0.116	0.735

Scale four T0	21.371 (3.398)	18.538 (3.356)	41	6.953	0.012

Scale four T1	21.296 (3.801)	19.461 (2.846)	42	1.568	0.218

Scale one. Attitude towards culturally appropriate care (5 = not at all important, 25 = extremely important)

Scale two. Experienced barriers towards culturally appropriate healthcare in general (4 = never barriers, 16 = always barriers)

Scale three. Experienced barriers towards culturally appropriate cardiovascular care and education (4 = no barriers, 16 = big barriers)

Scale four. Self-reported actions in delivering culturally appropriate care (7 = no actions, 28 = always actions)

*After correction for variable 'scale four T0' on confounding effects for the relationship between the intervention and variable 'scale one T1', a significant (p = 0.013) effect remains for the intervention on 'scale one T1'

## Discussion

We described a pilot study of an intervention to assist healthcare providers in delivering CAHE. Inspired by evidence from studies on professional behaviour change [28,29], the intervention consisted of multiple components: tools for CAHE that complemented an existing digital protocol for hypertension care, training, and feedback possibilities. Moreover, the content of the tools and the supportive interventions were aimed at removing previously observed barriers that may impede CAHE--a negative attitude towards culturally appropriate care and/or insufficient competence to implement it.

The results revealed that healthcare professionals who participated in the intervention considered it more important to address the patient's culture when delivering care than they had before the intervention. The current intervention did not influence experienced barriers and self-reported behaviour with regard to culturally appropriate care delivery.

The absolute value of the observed differences was modest, so the results should be interpreted with care. Nevertheless, they suggest that the intervention has been successful in eliciting attitude change among healthcare providers. In the light of the theories of professional behaviour change [37], we may conclude that the intervention has specifically contributed to the acceptance of change. This is an important condition for the next stages of change--actual change and maintenance.

Some limitations may have influenced our results. First, only 49% of the participants in the control group responded to both questionnaires, as compared to 68% in the intervention group. An analysis of the response rates reveals that 12 of the 35 participants in the control group (34%) did not return the questionnaire at T0. Of the remaining group, six people (26%) did not return the questionnaire at T1. Possibly, people in the control group were less motivated to fill out the questionnaire than those in the intervention group because they might not have perceived how this could benefit them. More observations in the control group would have increased the chance of finding significant differences on three of the scales. Second, the intervention group consisted of healthcare providers from PCHCs that had taken part in focus groups on delivering culturally appropriate care in our previous study [27]. This may explain the baseline scores of the group on scale four, the self-reported actions in the intervention group, leaving only limited room for improvement. However, a more in-depth understanding of experienced barriers to the application of the tools is needed. Third, we studied PCHCs that belong to the same primary healthcare consortium. Healthcare professionals from these PCHCs meet regularly in joint consortium meetings, thus contamination cannot be ruled out. Randomised study designs may be a better option for evaluating the true effect of an intervention, even in pilot studies. However, it should be acknowledged that randomised designs are not always possible in routine clinical practice because of organisational or ethical impediments. Moreover, even with randomised designs contamination can not always be prevented [38]. Fourth, in order to measure the attitudes, competence, and behaviour of the study population, we adapted an instrument standardised for measuring cultural competence among resident physicians in the USA [35]. A drawback of this instrument is that the questions were not always appropriate for NPs and GP assistants. Moreover, they were rather general and not specifically tailored to the objectives of the intervention. In future studies, other evaluation instruments that are more closely tailored to the specific objectives of the intervention may be considered.

There is an urgent need to improve hypertension education directed at ethnic minority populations of African origin [2,7,9]. Interventions to increase the cultural competence of hypertension care providers are a first step towards this end [25]. Multi-component interventions including information, education, and support are most likely to elicit innovations among professionals [28]. Our intervention is the first clearly described multi-component intervention specifically designed to stimulate cultural competence in hypertension educators. Before the clinical significance of interventions in healthcare can be tested successfully, iterative approaches are needed to study any potential barriers to implementation of the intervention [39]. This pilot study provides preliminary evidence that our intervention may positively influence attitudes with regard to the delivery of culturally appropriate hypertension care. Positive attitudes are an important condition for the uptake of new approaches in practice. As a next step our research group will make a qualitative assessment of organisational factors that may have hampered or facilitated the use of the new tools in practice. The results of these studies will then be used in the design of a subsequent study that aims to measure the effect of the intervention on blood pressure control and treatment adherence in patients [40].

## Competing interests

The authors declare that they have no competing interests.

## Authors' contributions

EB and JH designed the study. EB and JH developed the intervention and the questionnaire in dialogue with PB, KS, and other members of the research group. EB and JM analysed the data in dialogue with PB, JH, and KS. EB and JH wrote the paper. PB, JM, and KS commented on various draft versions of the manuscript. All authors read and approved the final manuscript.
